# Contribution of vascular risk factors to the relationship between ADHD symptoms and cognition in adults and seniors

**DOI:** 10.1038/s41598-021-03782-y

**Published:** 2021-12-20

**Authors:** Brandy L. Callahan, André Plamondon, Sascha Gill, Zahinoor Ismail

**Affiliations:** 1 Department of Psychology, University of Calgary, 2500 University Drive NW, Calgary, AB T2N 1N4 Canada; 2 Hotchkiss Brain Institute, Calgary, AB Canada; 3 Department of Educational Fundamentals and Practices, Laval University, Quebec, QC Canada; 4 Department of Applied Psychology and Human Development, University of Toronto, Toronto, ON Canada; 5 Department of Clinical Neurosciences, University of Calgary, Calgary, AB Canada; 6 Departments of Psychiatry and Community Health Science, University of Calgary, Calgary, AB Canada; 7 O’Brien Institute for Public Health, University of Calgary, Calgary, AB Canada

**Keywords:** ADHD, Human behaviour

## Abstract

Symptoms of attention-deficit/hyperactivity disorder (ADHD) in childhood have been found to be predictive of compromised cognitive function, and possibly even dementia, in later adulthood. This study aimed to test vascular risk as a hypothesized moderator or mediator of this association, because individuals with elevated ADHD symptoms frequently have comorbid vascular disease or risk factors which are recognized to contribute to later-life cognitive decline. Data from 1,092 adults aged 18–85 were drawn from the Enhanced Nathan Kline Institute Rockland Sample. Childhood ADHD symptoms (assessed using the Adult ADHD Clinical Diagnostic Scale) were assessed as predictors of cognitive functioning in adulthood (assessed using subtests from the University of Pennsylvania Computerized Neurocognitive Battery, the Delis-Kaplan Executive Functioning System, and the Wechsler Memory Scale). Vascular risk factors (including diabetes, tobacco use, obesity, hypertension, and hypercholesterolemia) were tested as both a moderator and mediator of this relationship. Childhood ADHD symptoms and vascular risk factors were both independently associated with later-life cognition, but vascular risk was not a significant moderator or mediator of relationships between ADHD symptoms and cognition in statistical models. Results from this large community sample suggest that the relationship between ADHD symptoms and cognition is not accounted for by vascular risk. This question should also be investigated in clinical samples.

## Introduction

Persistent symptoms of attention-deficit/hyperactivity disorder (ADHD) affect at least 3% of adults and seniors^1,2^. Its cognitive features (inattention, distractibility, impulsivity) tend to be associated with disruptions in executive functions (i.e., complex regulatory processes controlled in part by the frontal lobes)^3,4^. For example, increased ADHD symptoms in older adults, assessed as a continuous variable using the Conners Adult ADHD Rating Scale, have been linked to lower performance on tasks of working memory^5^ and retrieval^6^, as well as slowed reaction time^6^. This aligns with findings of compromised frontal lobe integrity in ADHD^7^ which, in older adults, may be compounded by the known deleterious effects of aging on frontal brain regions and processes^8,9^.

ADHD symptoms have recently been linked to accelerated cognitive decline later in life. Adults with a history of clinically-diagnosed ADHD may have higher likelihood of developing dementia than do adults without ADHD^10–12^. Similarly, individuals with Lewy body dementia retrospectively report more severe ADHD symptoms in childhood than do healthy controls^13^. The mechanistic processes underlying this proposed relationship are unknown. One possibility is that ADHD symptoms may increase risk for later-life cognitive impairment by promoting the accumulation of brain health-compromising factors and behaviors throughout adulthood^14^. Vascular risk factors, as well as health behaviors that jeopardize vascular health, have been associated with cognitive performance across numerous studies. Diabetes^15–19^, tobacco use^18,20^, obesity^21–23^, hypertension^17,18,24–26^, and hypercholesterolemia^18,27^ have all been linked to lower cognitive performance in older adults. These factors seem to have particular relevance for executive functions, as aggregate scores for vascular risk (e.g., hypertension, diabetes, and smoking) relate negatively to global measures of executive function^28,29^ as well as specific measures of switching and working memory^30^.

It is now well-recognized that ADHD symptoms are associated with increased risk of vascular medical comorbidities and engagement in harmful behaviors that may cumulatively compromise cerebrovascular and cognitive health throughout adulthood^31^. For instance, young people with ADHD are more likely than those without to develop diabetes later in life^12,32^, and those with high levels of ADHD symptoms are more prone to heavy daily cigarette smoking than those with low symptom levels^33^. ADHD symptoms have been robustly linked to later-life risk for overweight and obesity^12,34^, which may contribute to a secondary association between ADHD symptoms and hypertension in adults^35^. ADHD symptomatology has also been linked to higher blood concentrations of low-density lipoprotein (LDL) cholesterol^36^, which contributes to atherosclerosis and vascular disease with age^37^. In non-ADHD psychiatric samples, these comorbidities and health behaviors have been shown to account for a substantial proportion of variance in cognitive performance^38^.This relationship has not been tested in ADHD, but raises the possibility that impairments in executive functioning and potential accelerated cognitive decline and dementia risk in ADHD may be due in part to the disorder’s association with vascular risk factors. A corollary to this hypothesis is that active vascular risk management may alleviate some of the cognitive difficulties associated with ADHD symptoms.

In light of the known adverse cognitive effects of vascular risk factors in epidemiological studies of adults and seniors, and considering their association with symptoms of ADHD, this study’s objective is to investigate whether previously-reported associations between ADHD symptoms and cognitive impairment^10,11,13^ may be driven by vascular risk factors (diabetes, smoking, obesity, hypertension, and hypercholesterolemia). We hypothesize that the relationship between ADHD symptoms and cognitive performance will be mediated or moderated by these factors, and we focus on frontal/executive aspects of cognitive performance (processing speed, reaction time, working memory, cognitive flexibility, and inhibitory control) because these are the most associated with ADHD symptomatology in adults and have been robustly linked to physical health, as described above. We also focus on ADHD symptoms in childhood as they are more likely to have preceded, rather than followed, the presence of vascular risk factors.

## Materials and methods

### Participants

This study used data from the Enhanced Nathan Kline Institute Rockland Sample (NKI-RS)^39^, a lifespan, cross-sectional community sample of individuals aged 6–85 years. Only data from participants 18 or older were retained for the present study (N = 1,092). The NKI-RS has institutional ethical approval at the Nathan Kline Institute (#226,781 and #239,708) and at Montclair State University (#000983A and #000983B), all participants within this sample provided written informed consent to participate, and all phenotypic protocols developed by the Child Mind Institute’s Scientific Research Council were followed^39^.

### Materials

All data described below were collected within the scope of a larger protocol administered over two days^39^.

#### ADHD variables

ADHD symptoms were assessed using the Adult ADHD Clinical Diagnostic Scale (ACDS)^40^.This clinician-administered interview retrospectively assesses childhood ADHD in adult participants, and the interview then follows with an expanded assessment of recent symptomology (i.e., past 6 months). Although childhood symptoms (averaged across items) were the main focus of the current study, a current symptoms score was also computed (averaged across items) to examine possible associations with outcomes in an exploratory fashion.

#### Vascular health variables

Consistent with prior studies of vascular risk and cognition eg., ^41^, the following risk factors were included in the present study. Diabetes was defined as either a self-reported diagnosis of diabetes on a medical history questionnaire, or a fasting glucose level of ≥ 140.0 mg/dL on bloodwork. Tobacco use in the past two years (yes/no) was ascertained using the Fagerstrom Test for Nicotine Dependence^42^. Obesity was quantified as body mass index (BMI) > 30 kg/m^2^. Participants with systolic blood pressure > 140.0 mm/Hg were considered hypertensive. Non-high-density lipoprotein (non-HDL) was isolated from total cholesterol volumes to define hypercholesterolemia, as recommended in prior literature^43^, by subtracting HDL from total cholesterol. Values > 158 mg/dL were considered to reflect hypercholesterolemia.

#### Cognitive variables

Cognitive performance was assessed using subtests of the University of Pennsylvania Computerized Neurocognitive Battery (CNB)^44^, the Delis-Kaplan Executive Functioning System (D-KEFS)^45^, and the Wechsler Memory Scale-Revised (WMS-R)^46^. Domains assessed included switching (i.e., shifting flexibly between tasks)^47^, working memory (i.e., temporary storage and manipulation of information in a complex task)^48^, reaction time (i.e., decision-making latencies to respond to visual stimuli)^49^, and processing speed (i.e., response latencies to simple cognitive tasks requiring little to no complex processing)^50^. A detailed description of the variables, along with any transformations applied to the data, appears as Supplementary Information.

### Statistical analyses

Analyses were performed in Mplus 8.1. Since some variables were treated as categorical, we used the Weighted Least Square—Means and Variance adjusted (WLSMV) estimator. The WLSMV estimator includes participants with missing data and provides consistent estimates when the data is missing-at-random conditional on covariates^51^. Missing data in the predictors was dealt with by imputing 20 datasets^52^. In structural equation modeling, model fit can be assessed using various indexes^53,54^. The χ^2^ is a measure of exact fit and should ideally be small and non-significant. It is often significant and other fit indices are often used. The Comparative Fit Index (CFI) and the Tucker-Lewis Index (TLI) are measures of relative fit. Values above 0.90 and 0.95 indicate good excellent fit, respectively. The Root Mean Square Error of Approximation should be small, with values below 0.08 and 0.05 indicating good and excellent fit, respectively.

Consistent with recent models of executive function^3,55^ we used a bifactor model to capture three components of executive function (EF). This model is a bifactor S-1 model^56^, which comprises a general factor as well as s-1 specific factors. Here, this implies that two out of the three components of EF have a specific factor. The component without a specific factor (i.e., inhibition) can be interpreted as a reference component that accounts for variance in other components. Its variance is captured in the general factor (gEF), and the variance it shares with the other two EF components is absorbed by the general factor. As a result, the two specific factors can be interpreted as residual variance shared by indicators after accounting for the variance of the reference component (i.e., inhibition). The model therefore has the following factors: (1) a general EF factor (gEF) capturing inhibition as well as its covariance with all other EF indicators; (2) a specific Switching factor (sSwitch) capturing variance shared by indicators of switching over and above what was accounted for by gEF; 3) a specific Working Memory factor (sWM) capturing variance shared by indicators of working memory, over and above what was accounted for by gEF.

To ensure that the gEF, sSwitch and sWM factors captured independent sources of variance, they were not allowed to correlate by statistically constraining their covariance to zero (i.e., they were orthogonal). We also modeled a Reaction Time factor (RT) and a Processing Speed factor (PS), both of which were allowed to correlate with each other and with the three components of executive function. Finally, we included a method factor capturing variance shared by tasks measured in the CNB battery, because we expected a priori that tasks from within the same battery (i.e., D-KEFS vs. CNB) would be more correlated with each other than with tasks from a different battery. Thus, tasks from a specific battery would share common variance that is not shared with tasks from another battery; this variance is assumed to be a methodological artifact. The inclusion of latent factors to account for this kind of artifact is common when using multi-trait, multi-method data^57^. Although it is theoretically possible to include a method factor for each assessment battery, this strategy often results in statistical problems such as lack of convergence, as was the case in our data. A common solution consists of excluding the method factor for one assessment battery; this was the approach used here. More specifically, we created a latent factor to capture variance that was specific to the CNP battery. Thus, its variance was orthogonal (i.e., uncorrelated) with all other latent factors. This CNP latent factor included loading on the six CNP tasks. All six loadings were statistically significant, indicating that these tasks indeed shared common variance that was not accounted for by the other factors.

**Figure 1:**
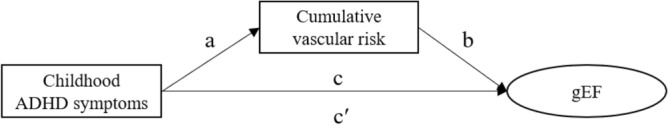
Conceptual model depicting the mediating role of cumulative vascular risk in the association between childhood ADHD symptoms and gEF. *Notes*. The indirect effect of ADHD symptoms on gEF via cumulative vascular risk is calculated by multiplying the “a” and “b” paths. The direct effect (c’) represents the effect of childhood ADHD symptoms on gEF after controlling for cumulative vascular risk. The total effect (c path) is the sum of the indirect (a*b) and direct (c’) effects. The same model is applied for each cognitive outcome.

**Figure 2:**
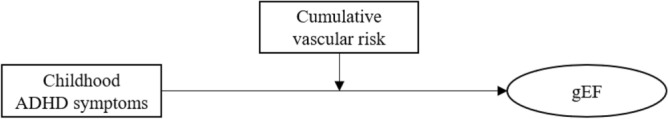
Conceptual model of the moderating role of cumulative vascular risk. *Note*. The association between childhood ADHD symptoms and gEF varies as a function of the level of cumulative vascular risk. The same model is applied to each cognitive outcome.

Our analyses were performed in four steps. First, we fitted a measurement model with only the outcomes (i.e., EF, RT and PS) to ensure that the hypothesized factor structure adequately fit the data. Second, we documented the associations between childhood ADHD symptoms and vascular risk factors by running an analysis where these were entered simultaneously as predictors of cognitive outcomes using multiple linear regressions. Third, we tested whether cumulative vascular risk mediated the association between ADHD symptoms and cognitive outcomes (Fig. 1). In other words, we aimed to see if the association between ADHD symptoms and cognition was best accounted for by vascular risk severity. For that purpose, we ran a series of structural equation models to test whether ADHD symptoms were associated indirectly with EF, RT and PS via vascular risk factors. The significance of the indirect effect was tested using 1000 bootstrap samples. Finally, we tested whether cumulative vascular risk moderated the association between childhood ADHD symptoms and cognitive outcomes (Fig. 2). In other words, we wanted to see if the strength of the association between ADHD symptoms and cognition varied as a function of vascular risk severity. Age was entered into the regressions as a categorical factor (18–65 years, > 65 years) to mitigate bias introduced by there being very young participants in the sample who were less likely to have cardiovascular risk factors. The age distribution in NKI-RS is bimodal, with peaks around 18 and around 60, and comparatively fewer middle-aged participants; creating an 18–65 group ensured sufficient heterogeneity within this group in terms of age and health risks.

Because age contributes independently to declines in executive functioning^58^ and vascular risk^59^, we also considered the moderating effect of age on these factors by conducting analyses testing whether age predicted either the cumulative risk score or executive functioning, including an interaction between the predictors and age as a categorical factor.

## Results

### Participants

Participant characteristics are reported in Table 1. The sample was 63.8% female and 75.7% White, with an average age of 47.2 years (*SD* = 18.0) and an average education of 15.5 years (*SD* = 2.3). Childhood ADHD symptom scores within the sample ranged from 0 to 30 (*M* = 2.0, *SD* = 4.0), and 50 participants (4.6%) met diagnostic criteria for one of the ADHD subtypes (22 inattentive, 5 hyperactive/impulsive, 9 combined, and 14 not otherwise specified) as determined by an NKI-RS clinician during assessment. Fifteen participants in the sample (1.4%) were taking stimulant medications at the time of testing (amphetamine/dextroamphetamine in six cases, methylphenidate in five, and lisdexamfetamine in four). The two most frequent vascular risk factors in the sample were obesity and hypercholesterolemia, observed in 30.5% and 23.9% of cases respectively. Most people had no vascular risk factors (42.9%), fewer people had only one (35.3%) or two risk factors (17.6%), and a small proportion of people had three or more risk factors (4.2%).

**Table 1:** Characteristics of the study sample.

Age (mean years, SD)	47.2 (18.0)
Sex (% female)	63.8%
Education (mean years, SD)	15.5 (2.3)
**Race**	
American Indian or Native Alaskan	0.9%
Asian	4.9%
Black or African American	15.5%
Native Hawaiian or Other Pacific Islander	0.5%
White	75.7%
Other Race	2.4%
Clinical diagnosis of ADHD (determined by NKI-RS clinicians)	4.6%
**Vascular risk factors**	
Diabetes (self-reported, or fasting glucose level ≥ 140.0 mg/dL)	5.9%
Tobacco use in last 2 years (self-reported)	12.7%
Obesity (BMI > 30 kg/m^2^)	30.5%
Hypertension (systolic blood pressure > 140.0 mm/Hg)	9.4%
Hypercholesterolemia (non-HDL cholesterol > 158 mg/dL)	23.9%
**Number of vascular risk factors**	
0	42.9%
1	35.3%
2	17.6%
3	3.6%
4	0.5%
5	0.1%

ADHD: attention-deficit/hyperactivity disorder. BMI: body mass index. gEF: general executive factor. HDL: high density lipoprotein. NKI-RS: Nathan Kline Institute Rockland Sample. PS: processing speed factor. RT: reaction time factor. SD: standard deviation. sSwitch: Switching factor. sWM: working memory factor.

### Measurement model

**Figure 3:**
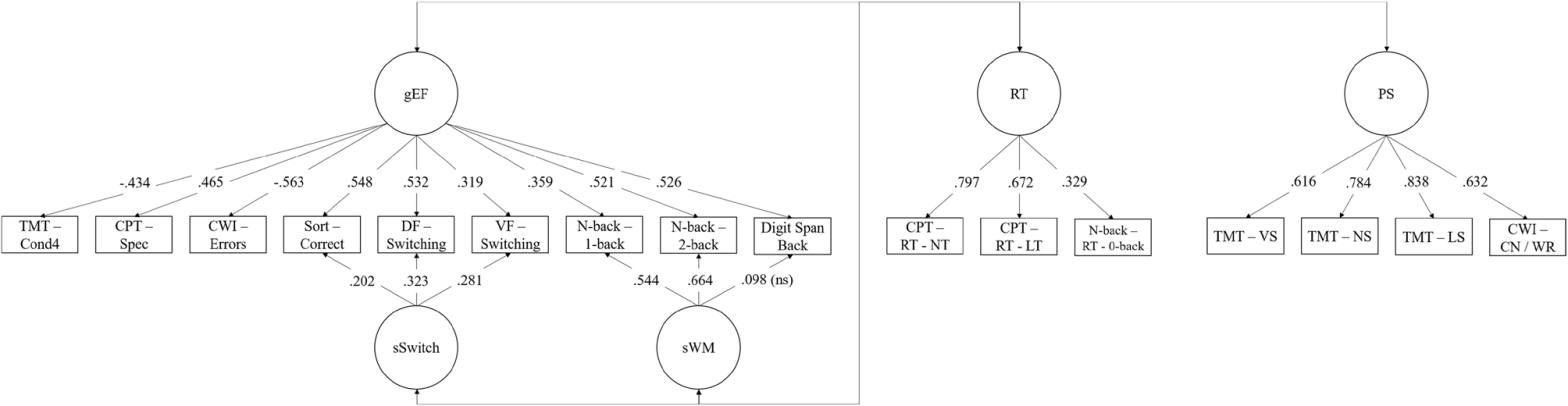
Confirmatory factor analysis of the cognitive outcomes with standardized factor loadings. *Notes*. Double-headed arrows represent correlations between latent factors﻿. The methodological factor capturing covariance between indicators measured in the Pennsylvania Computerized Neurocognitive Battery (CNB) battery is not depicted for the sake of clarity. Cond4 = Condition 4. CPT—RT-LT = Continuous Performance Test reaction time on letter trials. CPT—RT-NT = Continuous Performance Test reaction time on number trials. CWI—CN/WR = Color Word Interference color naming/word reading. DF = Design Fluency. gEF = General executive function. TMT—LS = Trail Making Test letter sequencing. TMT—NS = Trail Making Test number sequencing. TMT-VS = Trail Making Test visual scanning. PS = Processing speed. RT = Reaction time. Sort = Sorting. Spec = Specificity. VF = Verbal fluency. WM = Working memory.

The fit of the model was good to very good, χ^2^ (85) = 312.037, *p* < 0.0001, CFI = 0.951, TLI = 0.931, RMSEA = 0.050 (Fig. 3). All factor loadings were significant except one (digit span backward onto sWM), showing that indicators loaded well onto their hypothesized factor. RT and PS were associated with all EF latent factors, except for RT which was not associated with sWM. In terms of effect size, gEF showed medium to large associations with RT and PS (*r* = -0.28, *p* < 0.001 and *r* = 0.45, *p* < 0.001), sWM showed small associations with both RT and PS (*r* = 0.08, *p* > 0.05 and *r* = 0.19, *p* < 0.001), and sSwitch showed large associations with RT and PS (*r* = -0.46, *p* < 0.001 and *r* = -0.87, *p* < 0.001). RT and PS were also significantly associated (*r* = 0.48, *p* < 0.001).

### Associations between each risk factor and cognitive measures

Inspection of associations between ADHD symptoms and EF revealed that childhood ADHD symptoms were correlated with gEF (*r* = -0.10, *p* = 0.018) and sWM (*r* = 0.10, *p* = 0.025), but there were no significant associations between *current* ADHD symptoms and any variable. Thus, there were differential associations despite the fact that childhood and current ADHD symptoms were moderately associated (*r* = 0.53, *p* < 0.001). Given this finding, the following analyses were performed using childhood ADHD symptoms unless otherwise noted.

To investigate associations between individual risk factors and cognitive measures, all risk factors were entered simultaneously as predictors of each of the five cognitive outcomes in multiple linear regressions. These effects are reported in Table 2. For gEF, there was a medium negative effect of age and smoking, as well as a small negative effect of childhood ADHD symptoms, obesity, and hypertension. The only significant effect for sWM was a small positive effect of childhood ADHD symptoms. For sSwitch, there was a large negative effect of age, as well as a moderate positive effect of being female. For RT, there was a medium negative effect of age, a small negative effect of being female, and a medium negative effect of diabetes. For PS, there was a large negative effect of age, a medium negative effect of hypertension and a small negative effect of smoking (because higher RT and PS scores represent worse performance, positive effect sizes in Tables 2 and 3 represent deleterious effects on cognition).

**Table 2:** Standardized effects of individual risk factors predicting each outcome entered simultaneously in multiple linear regressions.

	gEF		sWM		sSwitch		RT		PS	
	β	*p* value	β	*p* value	β	*p* value	β	*p* value	β	*p* value
Age (≥ 65 years)	−.50*	.000	.03	.845	−1.07*	.000	.59*	.000	1.10*	.000
Sex (female)	−.06	.523	−.10	.363	.44*	.006	.20*	.020	−.04	.554
Childhood ADHD symptoms	−.11*	.008	.10*	.033	.06	.398	.02	.722	.01	.706
**Vascular risk factors**										
Diabetes	−.04	.836	−.20	.307	−.46	.178	.43*	.011	.26	.052
Smoking	−.51*	.000	−.14	.380	.03	.892	.07	.559	.25*	.011
Obesity	−.32*	.000	.22	.058	.24	.151	.02	.791	.10	.155
Hypertension	−.35*	.010	−.07	.659	−.19	.481	.23	.155	.41*	.000
Hypercholesterolemia	.15	.137	−.09	.441	-.19	.284	−.03	.759	.04	.611

*p < .05. ADHD: ttention-deficit/hyperactivity disorder. gEF: general executive factor. PS: processing speed factor. RT: reaction time factor. sSwitch: Switching factor. sWM: working memory factor. All effects (except ADHD symptoms) are standardized using only the outcome variance (STDY in MPlus) and reflect the increase in standard deviation of the outcome for an increase of one in the raw metric of the predictor. Effect sizes may therefore be interpreted using Cohen’s d (small: d = .20, medium: d = .50, large: d = .80). ADHD symptoms were continuous so their effects were standardized using for the predictor and the outcome variances (STDYX in MPlus). Effect sizes may therefore be interpreted using Cohen’s *r* (small: *r* = .10, medium: *r* = .30, large: *r* = .50). Because higher RT and PS scores represent worse performance, positive effects represent deleterious effects on cognition.

**Table 3:** Standardized effects of individual risk factors and cumulative vascular risk factors predicting each outcome entered simultaneously in multiple linear regressions.

	gEF		sWM		sSwitch		RT		PS	
	β	*p* value	β	*p* value	β	*p* value	β	*p* value	β	*p* value
Age (≥ 65 years)	−.46*	.000	.04	.781	−1.18*	.000	.62*	.000	1.13*	.000
Sex (female)	−.02	.840	−.10	.363	.42*	.008	.19*	.026	−.05	.411
Childhood ADHD symptoms	−.12*	.005	.11*	.029	.06	.354	.01	.812	.01	.718
**Vascular risk factors**										
One risk factor	−.22*	.024	−.15	.218	−.01	.970	.14	.130	.14	.053
Two risk factors	−.35*	.003	.04	.783	−.02	.910	.10	.405	.27*	.003
≥ Three risk factors	−.78*	.000	.03	.904	−.18	.664	.37	.082	.62*	.000

*p < .05. ADHD: ttention-deficit/hyperactivity disorder. gEF: general executive factor. PS: processing speed factor. RT: reaction time factor. sSwitch: Switching factor. sWM: working memory factor. All effects (except ADHD symptoms) are standardized using only the outcome variance (STDY in MPlus) and reflect the increase in standard deviation of the outcome for an increase of one in the raw metric of the predictor. Effect sizes may therefore be interpreted using Cohen’s d (small: d = .20, medium: d = .50, large: d = .80). ADHD symptoms were continuous so their effects were standardized using for the predictor and the outcome variances (STDYX in MPlus). Effect sizes may therefore be interpreted using Cohen’s *r* (small: *r* = .10, medium: *r* = .30, large: *r* = .50). Because higher RT and PS scores represent worse performance, positive effects represent deleterious effects on cognition.

### Effect of cumulative vascular risk

To then test whether vascular risk factors predict cognitive functioning according to a cumulative model, we computed a cumulative vascular risk score by creating a sum of the five risk factors, regardless of their nature. As there were few people with three or more risk factors (see above), we recoded the cumulative risk score into four categories: 0, 1, 2 and ≥ 3 risk factors.

**Figure 4:**
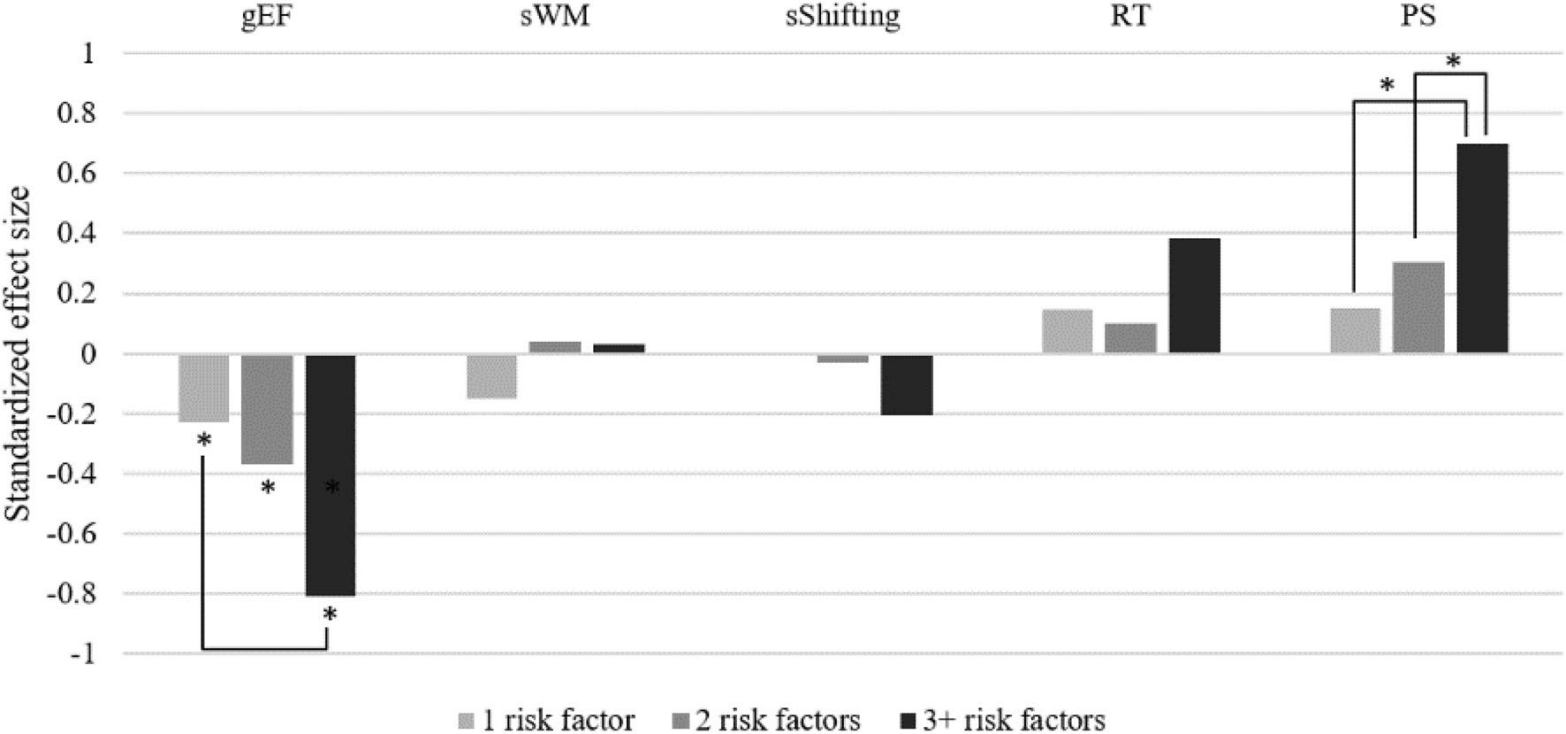
Associations between the number of vascular risk factors and cognitive functioning. *Notes*. Effects correspond to those from the model estimated and reported in Table 3. All effects are standardized using only the outcome variance (STDY in MPlus) and reflect the increase in standard deviation of the outcome for an increase of one in the raw metric of the predictor. Effect sizes may therefore be interpreted using Cohen’s *d* (small: *d* = .20, medium: *d* = .50, large: *d* = .80). gEF: general executive factor. PS: processing speed factor. RT: reaction time factor. sSwitch: Switching factor. sWM: working memory factor.

This cumulative score was used to predict cognitive functioning while also controlling for sex, age and childhood ADHD symptoms (see Table 3 and Fig. 4). For gEF, there was a medium negative effect of age, as well as a small negative effect of childhood ADHD symptoms. Vascular risk factors predicted significantly poorer gEF, with increasing number of risk factors showing increasingly larger effects. The only significant effect for sWM was a small positive effect of childhood ADHD symptoms. For sSwitch, there was a large negative effect of age, as well as a medium positive effect of being female. For RT, there was a medium to large negative effect of age, and a small negative effect of being female. For PS, there was a large negative effect of age, as well as a negative effect of vascular risk, with 2 and ≥ 3 risk factors being significantly associated with poorer processing speed.

Because vascular risk factors were associated with both gEF and PS, we tested whether the link between vascular risk factors and gEF was accounted for by their effects on processing speed. We ran the model reported in Table 3 but regressed gEF on PS to see whether the effect of vascular risk factors on gEF would diminish. The direct effect of PS on gEF was moderate to large in magnitude (β = 0.424, *p* = 0.002) and the magnitude of the effects of the number of risk factors was reduced by about half (1 risk factor: β = 0.152, *p* = 0.107, 2 risk factors: β = 0.236, *p* = 0.033, ≥ 3 risk factors: β = 0.496, *p* = 0.013). Although vascular risk factors were indirectly associated to gEF via PS (total effect: b = 0.232, *p* = 0.000, indirect effect: 0.077, *p* = 0.011), accounting for about a third of this association (0.077/0.232 = 33.19%), ADHD symptoms were not associated with gEF via PS (total effect: b = 0.933, *p* = 0.014, indirect effect: 0.035, *p* = 0.786).

### Mediating role of cumulative vascular risk

Contrary to our expectations, there was no association between ADHD symptoms in childhood and cumulative vascular risk (path *a* in the model shown in Fig. 1) after accounting for age and sex, OR = 1.378, 95% CI [0.684, 2.772]. Consistent with the fact that path *a* of the mediation model was not significant, we found no evidence that vascular risk factors mediated the link between ADHD symptoms in childhood and any of the cognitive outcomes.

### Moderating role of cumulative vascular risk

We then tested whether the association between ADHD symptoms and cognition was moderated by cumulative vascular risk, as this does not rely on the assumption that ADHD symptoms and cumulative vascular risk are associated. Also contrary to our hypothesis, we found no evidence that childhood ADHD symptoms moderated the effect of the number of vascular risk factors (detailed results not reported).

### Moderating effect of age

There was no significant interaction between age categories (above/below age 65) and ADHD symptoms in predicting cumulative risk score (b = 0.227, *p* = 0.791). In predicting the EF components, we looked at the interactions between age and either cumulative risk or ADHD symptoms. None of the interactions were significant in predicting gEF, sWM or sShifting.

### Exploratory analyses

We considered the possibility that ADHD symptom subtypes may have differential associations with health outcomes (e.g., perhaps the hyperactive/impulsive subtype is at higher risk for vascular risk factors and poor self care relative to the inattentive subtype). To explore the possibility that meaningful effects of individual symptom subtypes may have been lost in the consolidation of subtypes into a global ADHD measure, we reconducted the above mediation and moderation analyses using inattentive and hyperactive/impulsive symptoms in separate models. All results remained non-significant (detailed results not reported).

## Discussion

Using a large community sample, this study aimed to investigate whether the relationship between ADHD symptoms and frontally mediated aspects of cognitive performance could be accounted for by vascular risk factors, which have been commonly reported in ADHD^31^ and are recognized to increase risk for cognitive impairment in adults^18,41,60–62^. Although we found that childhood ADHD symptoms and current vascular risk factors were independently associated with aspects of current cognitive performance, we found no evidence that vascular risk mediated or moderated the relationship between ADHD and cognition.

First, we found that childhood ADHD symptoms had only weak negative effects on current general executive functioning (path *c* in Fig. 2). Although this is contrary to historical conceptualizations of ADHD as primarily a dysexecutive disorder, there is growing recognition that executive dysfunction is not universal in ADHD^63^. These findings may also reflect community sampling within the NKI-RS, which resulted in relatively few (4.6%) cases who met diagnostic criteria for clinical ADHD. Surprisingly, we found small *positive* effects of childhood ADHD symptoms on the specific working memory factor, a finding which is particularly difficult to reconcile with prior reports of moderate-magnitude negative associations between ADHD symptoms and working memory performance in adults^64^. However, because we used a bifactor model, the specific WM and Switching factors are in fact *residuals,* as they exclude any variance shared with inhibition. Further, the sWM factor was also poorly captured in this sample, given that backwards digit span did not load significantly onto it. Given the factor loadings, the sWM factor captures shared variance by both n-back indicators, and it may thus be argued that this model is capturing task-specific variance rather than true working memory abilities.

Our results additionally revealed mild to moderate negative associations between vascular risk and aspects of cognitive function (path *b* in Fig. 2), in particular general executive abilities, processing speed and reaction time. The relationship between vascular disease burden and impairments in speeded responding is well documented e.g.,^65–70^ and is thought to be caused by damage to white matter fibre tracts within frontal-subcortical neuronal circuits^71^. Also consistent with previous research^72^, the deleterious effects of vascular risk factors on cognition were additive, with increasing number of risk factors showing increasingly larger effects.

The central question in this study was whether vascular risk factors explained a significant portion of the association between ADHD symptoms and cognitive outcomes. Contrary to our hypothesis, results from this sample demonstrated that the respective effects of ADHD symptoms and vascular risk factors on cognition are largely independent. After controlling for cumulative vascular risk, the direct effect of childhood ADHD symptoms on cognition remained significant, indicating that the association between ADHD symptoms and cognition is not accounted for by the presence or severity of vascular risk factors. At the same time, we found no evidence that childhood ADHD symptoms were linked to vascular health in adulthood in this sample (i.e., a non-significant path *a* in Fig. 2), which was relatively surprising considering others’ findings of heightened vascular risk in adults with significant childhood ADHD symptoms. Obesity appears to be the most consistent vascular-related comorbidity among adults with ADHD^73^. Other vascular risk factors are more debated, with some studies finding increased prevalence of comorbidities such as hypertension and diabetes^74^, and others reporting similar prevalence estimates of cardiovascular disease and most vascular risk markers between adults with and without ADHD^75^. Even upon testing a model which does not rely on the assumption that ADHD symptoms and vascular risk are associated (i.e., a moderation model), we again found no evidence that vascular risk influenced the direction or strength of the relationship between ADHD symptoms and cognitive outcomes.

These results tentatively suggest that prior reported associations between ADHD and cognitive impairment^10,11,13^ are not likely driven by vascular disease burden, although our cross-sectional sample composed primarily of non-clinical cases of ADHD precludes definitive conclusions about risk of cognitive *decline* in clinical ADHD. There is substantial evidence that midlife health factors influence cognitive decline several decades later^41,62^, and the dataset we used for this study unfortunately did not allow to account for temporal effects between vascular risk and cognition. We are aware of one study examining relationships between ADHD, cognitive outcomes and vascular risk factors longitudinally across a decade, which reported that any associations between a history of diagnosed ADHD and subsequent dementia are lost after controlling for diabetes and obesity^10^. This suggests that these vascular risk factors may account for considerable variance in the relationship between clinical ADHD and 10-year dementia risk; additional prospective longitudinal studies will be needed to validate these findings in adults across the ADHD symptom severity spectrum, with richer cognitive and behavioural outcomes. Further, there has been increasing recognition of other medical and physical conditions in ADHD^12,76^ that were not examined here but that may also contribute to cognitive changes into later adulthood, such as liver disease^77^, epilepsy^78^ and obstructive sleep apnea^79^. These conditions should be explored as potential mediators in future work. Similarly, given high rates of depression in ADHD^80,81^, and the links between vascular disease, depression, and impaired executive function^82,83^, it will be relevant to investigate the contributions of depression to cognitive decline in later-life ADHD. We are aware of at least one previous study reporting that ADHD symptoms were indirectly associated with cognition via depressive symptoms^6^, and it will be worthwhile to extend this to clinical ADHD cohorts and examine risk of dementia status.

In addition, NKI-RS constitutes a community sample, which includes very few cases of clinical ADHD (only 50 cases, or 4.6% of the sample) with even fewer of these in the older-adult range (10 cases, or 1% of the sample). It is possible that only individuals with very severe symptoms of ADHD experience significant vascular burden, potentially due to chronic stimulant medication use^84^, poor dietary and exercise habits^85^, or increased vascular inflammatory reactivity^86^, and adults over age 50 may be especially susceptible^74^. Our sample may have been underpowered to detect these effects due to few older adults with severe ADHD symptoms. Lastly, it may also be worthwhile for future work to examine the impacts of pharmacological management of ADHD in later life. Available medications include both stimulant and non-stimulant psychotropics, and each may differentially affect ADHD domains and quality of life^87^.

## Conclusion

In summary, results from this large community sample suggest that vascular risk does not play a significant role in explaining the relationship between milder, non-clinical childhood ADHD symptoms and cognition. This question should also be investigated in clinical samples of individuals with more severe ADHD symptomatology, for whom vascular health and cognition may be particularly compromised.

## Supplementary Information

Supplementary Information.
