# Supplementary material for: Contribution of vascular risk factors to the relationship between ADHD symptoms and cognition in adults and seniors
Source: Sci Rep. 2021 Dec 20;11:24276. doi: 10.1038/s41598-021-03782-y (PMC8688479; doi:10.1038/s41598-021-03782-y)
Supplement: Supplementary file 1 — Supplementary Information. [file 41598_2021_3782_MOESM1_ESM.docx]

Appendix. Cognitive variables used in statistical models

|  | NKI-RS variable name | Description | Transformation |
| --- | --- | --- | --- |
| General EF factor (gEF) | | | |
| TMT Switching | TMT_47 | D-KEFS Letter-Number Switching (all error types, total raw) | Data collapsed into categories* |
| CPT Specificity | CNP_0031 | Represents ([true negatives]/[true negatives+false positives]) responses on the CNB CPT | Cubed to deal with left skewness |
| CWI Errors | CWI_37  CWI_39 | D-KEFS CWI Inhibition (all error types, total raw)  D-DEFS CWI Inhibition/Switching (all error types, total raw) | Mean (CWI_37 CWI_39) |
| Specific switching factor (sSwitch) | | | |
| Sorting | SORT_42 | D-KEFS Sorting confirmed correct sorts (total raw) |  |
| Design Fluency Switching | DF_03 | D-KEFS Switching count correct (total raw) |  |
| Verbal Fluency Switching | VF_33 | D-KEFS Category Switching sum of the number of correct individual responses for 2 category trials (total raw) |  |
| Specific Working Memory factor (sWM) | | | |
| N-Back - 1-back | CNP_0042 | True positive responses for 1-back trials (total raw) on the N-Back task within CNB | Data collapsed into categories* |
| N-Back - 2-back | CNP_0045 | True positive responses for 2-back trials (total raw) on the N-Back task within CNB | Data collapsed into categories* |
| Digit Span Back | DSPAN_04 | WMS-R Digit Span Backward correct responses (total raw) | Data collapsed into categories* |
| Reaction Time factor (RT) | | | |
| CPT Number Trials | CNP_0013 | Median response time for short CPT true positive responses for number trials (milliseconds) on the N-Back task within CNB |  |
| CPT Letter Trials | CNP_0019 | Median response time for short CPT true positive responses for letter trials (milliseconds) on the N-Back task within CNB |  |
| N-Back - 0-Back | CNP_0041 | Median response time for correct 0-back trials (milliseconds) on the N-Back task within CNB |  |
| Processing Speed factor (PS) | | | |
| TMT Scanning | TMT_01 | D-KEFS Visual Scanning – Completion Time (seconds) | Log transformed |
| TMT Number Sequencing | TMT_02 | D-KEFS Number Sequencing - Completion Time (seconds) | Log transformed |
| TMT Letter Sequencing | TMT_03 | D-KEFS Letter Sequencing- Completion Time (seconds) | Log transformed |
| CWI Color Naming & Word Reading | CWI_03  CWI_06 | D-KEFS CWI Color Naming (total time to complete, seconds)  D-KEFS CWI Word Reading (total time to complete, seconds) | Log(mean(CWI_03 CWI_06)+1) |

*Notes.* CNB = Pennsylvania Computerized Neurocognitive Battery. CPT = Penn Continuous Performance Test. CWI = Color Word Interference. D-KEFS = Delis-Kaplan Executive Functioning System. NKI-RS = Nathan Kline Institute Rockland Sample. TMT = Trail Making Test. WMS-R = Wechsler Memory Scale-Revised. *Rare observations were collapsed to create fewer than 10 categories (needed to treat the data as categorical in Mplus).
